# Effect of vitamin D supplementation on incidence of bronchopulmonary dysplasia among preterm infants up to 36 weeks' gestational age

**DOI:** 10.1002/ncp.11323

**Published:** 2025-05-28

**Authors:** Tara Rebele, Corey Hawes, Stephani Johnson, Melanie Newkirk

**Affiliations:** ^1^ Department of Clinical and Preventive Nutrition Sciences, Rutgers The State University of New Jersey New Brunswick New Jersey USA; ^2^ Human Nutrition and Health BASF Corporation Florham Park New Jersey USA; ^3^ Kentucky Children's Hospital University of Kentucky Lexington Kentucky USA

**Keywords:** bronchopulmonary dysplasia, preterm infants, vitamin D supplement

## Abstract

Preterm infants, especially those born at a younger gestational age (GA), are at risk for developing bronchopulmonary dysplasia (BPD), which can lead to longer hospitalizations, chronic pulmonary morbidity, and mortality. Vitamin D plays a role in lung and immune system development, and deficiency at birth is associated with a greater incidence of BPD among preterm infants. The purpose of this literature review was to determine the impact of vitamin D supplementation on BPD incidence among preterm infants born ≤36 weeks GA. A literature search of the PubMed, CINAHL, SCOPUS, and Google Scholar databases was conducted searching for clinical studies published since 2014 that evaluated the effect of vitamin D supplementation on BPD incidence among preterm infants ≤36 weeks GA. We identified and reviewed six clinical studies published between 2014 and 2023, including a total of 545 preterm infants born 25–34 weeks GA. Vitamin D supplementation between 800–1000 IU/day was safe and effective in significantly improving vitamin D status and significantly reducing vitamin D deficiency; however, positive findings regarding the influence of vitamin D supplementation in reducing the incidence of BPD were not consistent. More research is needed in the form of well‐designed RCTs investigating the effect of vitamin D supplemented at 800–1000 IU compared with the standard 400 IU dose on the incidence of BPD as the primary outcome.

## INTRODUCTION

Bronchopulmonary dysplasia (BPD) is a pulmonary illness in preterm infants that is caused when the alveolar stage of lung development is disrupted because of premature birth, resulting in respiratory distress requiring ventilation support, ultimately persisting into a chronic condition.^1^ Although the exact etiology of BPD is still unknown, it is characterized by pulmonary inflammation and increased inflammatory cytokines and chemokines that interfere with lung maturation and tissue repair.^2^ BPD is the most common comorbidity experienced by preterm infants, with an estimated global incidence between 17% and 75% when based on the definition of requiring supplementation oxygen at 36 weeks postmenstrual age (PMA).^1^ This wide range of BPD incidence is attributed to variations between studies in sample size, gestational age (GA), birth weight (BW), and BPD diagnosis criteria, as well as whether or not data for nonsurviving infants with BPD were included.^1^ BPD is a leading cause of mortality among very preterm infants born <32 weeks GA and is associated with longer hospitalization, increased medical costs, need for supplemental oxygen therapy after discharge, risk for rehospitalization, and chronic lung disease extending throughout childhood.^1^, ^3^, ^4^, ^5^

The primary predictive risk factor for BPD is GA at birth^6^ and infants born between 24 to 32 weeks GA are at the greatest risk.^5^ Other risk factors include genetics, prenatal infections/inflammation, antenatal steroids, prenatal and postnatal nutrient deficiencies, and postnatal oxygen toxicity or lung injury from mechanical ventilation.^5^ Fetal lung development occurs in the first trimester during the fifth week of gestation, taking up to the first 3 years of life to fully mature.^7^ The stage of lung development and maturity of a neonate's lungs depends on their GA at birth, which may encompass the end of the canalicular phase when surfactant is first synthesized at 24 weeks GA, the saccular phase at 25–34 weeks GA, or the final alveolar phase.^7^ The canalicular phase is characterized by pulmonary epithelium cell differentiation and surfactant synthesis.^7^ Surfactant, a lipoprotein complex, is secreted by the type 2 (ATII) pneumocytes on the alveoli surface and reduces surface tension to promote improved gas exchange, inflammation modulation, and host defense mechanisms.^7^ Terminal saccules are developed during the saccular phase from 25 to 34 weeks GA,^6^, ^7^ and they begin to divide in the final alveolar phase from 36 weeks GA.^7^ During the alveolar phase, which continues through the first 3 years of life, the dividing saccules create a network of alveoli that results in a 20‐fold increase in the gas exchange surface area.^8^

The role of the fat‐soluble vitamin D in lung development is through its activity as a steroid hormone in stimulating the vitamin D receptors in the ATII cells to synthesize surfactant, as well as triggering alveolar cell proliferation and differentiation.^5^, ^7^ Additionally, vitamin D's antioxidative, antifibrotic, and anti‐inflammatory properties play a part in the pathogenesis of lung illness and injury through immunomodulation and the regulation of the synthesis and functioning of T cells.^7^ A recent systemic review and meta‐analysis revealed that the majority (70%) of preterm infants are born with vitamin D deficiency (VDD) and low serum circulation of 25‐hydroxyvitamin D (25[OH]D) and VDD at birth was significantly associated with the development of BPD among preterm infants.^9^ Daily vitamin D recommendations and the criteria for categorizing vitamin D deficiency, sufficiency, and toxicity from the American Academy of Pediatrics (AAP) the European Society of Pediatric Gastroenterology, Hepatology (ESPGHAN) Committee on Nutrition are shown in Table 1.^10^, ^13^ Both the AAP and ESPGHAN determine daily recommendations based on vitamin D intake that will support circulating 25(OH)D > 20 ng/ml, levels that prevent deficiency and support adequate calcium absorption and bone mineralization;^10^, ^11^, ^13^ however, the optimal daily dose of vitamin D to prevent BPD has not been elucidated.

**Table 1:** Daily vitamin D recommendations and criteria for deficiency, sufficiency, and toxicity.^10^, ^11^

Vitamin D	AAP	ESPGHAN
Recommended intake (IU/day)	400	400–700^a^
Serum vitamin D (ng/mL)^b^		
Deficiency	<20	<25
Sufficiency	>20	>50
Toxicity	>100	>120

Abbreviations: AAP, American Academy of Pediatrics; ESPGHAN, European Society of Pediatric Gastroenterology, Hepatology.

^a^ Max daily dose 1000 IU/D; reccomended dose range provides approximately 300–525, 400–700, and 600–1000 IU/day for infants weighing 750 g, 1,000 g, and 1,500 g, respectively^10^

^b^ 1 ng/ml vitamin D = 2.5 nmol/L^12^

Kumar et al.^14^ published a systemic review and meta‐analysis in 2020 that aimed to determine the effect of vitamin D supplements compared with placebo on various clinical outcomes, including BPD.^14^ Three randomized clinical trials (RCTs), including 2479 very low BW or preterm infants, were analyzed concluding, with low‐certainty evidence based on the results of one study by Fort et al.,^15^ that despite daily 200 or 800 IU vitamin D supplementation, the relative risk of developing BPD was 0.77 (95% CI: 0.47–1.27).^14^ Although the 2016 Fort et al.^15^ RCT evaluated the effect of 200 IU and 800 IU vitamin D on serum 25(OH)D levels compared with placebo among 100 preterm infants, the study was not powered to compare clinical outcomes, such as BPD, between groups.^15^ Therefore, the purpose of this literature review is to determine the impact of vitamin D supplementation on the incidence of BPD among preterm infants born ≤36 weeks GA.

## METHODS

In February 2024, a search was conducted of the PubMed, CINAHL, SCOPUS, and Google Scholar databases for studies published in English in the previous 10 years between 2014 and 2024 meeting the following inclusion criteria: (1) hospitalized preterm infants born ≤36 weeks GA, (2) intervention or treatment with vitamin D supplementation, and (3) data collected on BPD incidence. Search terms included preterm infants, premature infants, very low BW infants, extremely low BW infants, ELBW, vitamin D, vitamin D supplements, vitamin D dosage, vitamin D dose, cholecalciferol, calciferol, bronchopulmonary dysplasia, BPD, neonatal chronic lung disease, and CLD. Studies evaluating vitamin D status and BPD incidence but not investigating vitamin D–supplement dose and studies that evaluated vitamin D supplementation and respiratory illnesses other than BPD were excluded from this analysis.

**Figure 1:**
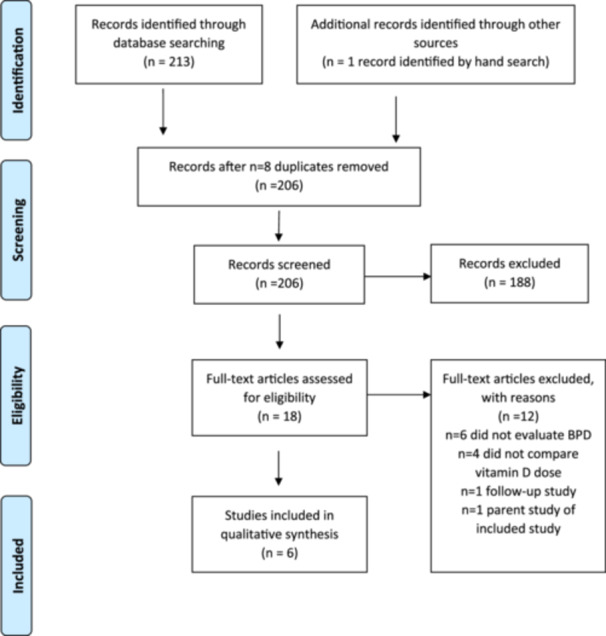
Flow diagram of the study selection process.

The initial database search, as presented in Figure 1, returned 213 records: eight duplicates were removed, 206 records were screened, and 17 full‐text articles were assessed for eligibility. Citation search identified one additional unique record for full‐text assessment. Twelve of the 18 full‐text articles assessed were excluded for not meeting the inclusion criteria (*n* = 6 no BPD data, *n* = 4 no vitamin D intervention, *n* = 1 follow‐up study, *n* = 1 parent study of an included study) and the remaining six studies were selected for inclusion in this review. The primary author graded the quality of each article according to the Academy of Nutrition and Dietetics Quality Criteria Checklist for Primary Research^16^ (Table 2) and all members of the research team reviewed and agreed on the quality grades for each article.

**Table 2:** Overview of study characteristics.

	Study design, quality grade^a^	GA & BW inclusion	Birth weight (g) & GA (wk) by treatment group mean (±SD)	Control vitamin D dose (*n*)	Experimental vitamin D dose(*n*)	Intervention initiation	Intervention duration	Study outcome measurements
Aristizabal et al., 2023 United States	Secondary data analysis of RCT^b^ +	≤28 wk	Placebo: 773.0 (±206.0), 25.0 (±1.0) 200 IU: 846.0 (±154.0), 26.0 (±1.0) 800 IU: 839.0 (±224.0), 25.0 (±1.0)	Placebo (*n* = 31)	200 IU (*n* = 19) 800 IU (*n* = 23)	Within 72 h of EN initiation	28 days	Serum 25(OH)D Rate of VDD^c^ Rate of BPD and predictive risk of BPD at 36 wk PMA or discharge
Ge et al., 2022 China	Prospective unblinded RCT +	<32 wk <1500 g	Control: 1290.0 (±170.0), 29.8 (±1.1) 800 IU: 1240.0 (±150.0), 29.2 (±1.4)	No vitamin D supplement (*n* = 55)^d^	800 IU (*n* = 57)	Within 48 h after birth	28 days	Serum 25 (OH)D BPD incidence
Elfarargy et al., 2022 Thailand	Prospective double‐blind RCT Ø	Premature infant with RD receiving MV FiO2 >21%	Placebo: 1914.2 (±92.0), 33.3 (±1.4) 800 IU: 1913.7 (±93.0), 33.4 (±1.3)	Placebo 2 ml distilled water (*n* = 50)	800 IU (*n* = 50)	Day 1 of NICU admission	14 days	Serum 25(OH)D Development of BPD
Cho et al., 2017 South Korea	Prospective cohort trial +	<1500 g	Total: 971.2 (±252.4), 27.1 (±2.5)	N/A	800 IU (*n* = 52)	Day 14	Until 36 wk PMA	Vitamin D status at 32 and 36 weeks PMA^e^
Bozkurt et al., 2017 Turkey	Prospective unblinded RCT +	≤32 wk	400 IU: 1242 (±230), 28.8 (±1.8) 800 IU: 1145 (±268), 29.0 (±1.9) 1000 IU: 1147 (±222), 28.7 (±1.9)	400 IU (*n* = 40)	800 IU (*n* = 41) 1000 IU (*n* = 40)	After reaching 75% of EN goal rate	Until 36 wk PMA	Serum 25 (OH)D Prevalence of VDD^c^ Prevalence of BPD
Natarajan et al., 2014 India	Prospective double‐blind RCT +	28–34 wk	400 IU: 1694.0 (±513.0), 32.5 (±1.8) 800 IU: 1655.0 (±411.0), 32.4 (±1.9)	400 IU (*n* = 42)	800 IU (*n* = 45)	After drawing baseline laboratory tests	Until 40 wk PMA	Serum 25(OH)D and VDD at 40 wk PMA^c^

Abbreviations: 25(OH)D, 25‐hydroxy vitamin D; BW, birthweight; BPD, bronchopulmonary dysplasia; CA, corrected age; EN, enteral nutrition; FiO2, fraction of inspired oxygen; GA, gestational age; IU, international units; MV, mechanical ventilation; NICU, neonatal intensive care unit; PMA, postmenstrual age; RCT, randomized control trial; RD, respiratory distress; wk, week; VDD, vitamin D deficiency.

^a^ Quality Grade definition according to the Academy of Nutrition and Dietetics Quality Criteria Checklist for Primary Research: + positive, − negative, Ø neutral.^16^

^b^ Parent study: Fort et al., 2014.^15^

^c^ Vitamin D deficiency serum 25(OH)D < 20 ng/ml.

^d^ Control intervention not described.

^e^ Vitamin D status serum 25(OH)D > 30 ng/ml= sufficiency and <10 ng/ml = deficiency.

## RESULTS

The six primary research studies selected for this review (Table 2) were published between 2014 and 2023, including two double‐blind RCTs,^17^, ^18^ two unblinded RCTs,^19^, ^20^ one prospective cohort study,^21^ and one secondary data analysis of an RCT.^22^ Outcomes data as shown in Table 3 were reported on a total of 545 preterm infants with GA ranging from 25 to 34 weeks.^17^, ^18^, ^19^, ^20^, ^21^, ^22^ Three studies specifically investigated vitamin D supplementation and incidence of BPD,^17^, ^19^, ^22^ whereas the other three studies examined the clinical efficacy of vitamin D supplementation and reported BPD incidence outcomes.^18^, ^20^, ^21^ All of the studies administered the daily vitamin D intervention doses via the enteral route; however, the intervention dose varied from 200 IU to 1000 IU.^17^, ^18^, ^19^, ^20^, ^21^, ^22^

**Table 3:** Vitamin D status and BPD outcomes before and after study intervention.

	Serum 25(OH)D mean (±SD) ng/ml		Rate of VDD^a^		
	Baseline	Post‐intervention	Baseline *n* (%)	Post‐intervention *n* (%)	BPD incidence *n* (%)
Aristizabal et al., 2023^b^	Control (*n* = 31): 18.7 (±12.9) 200 IU (*n* = 19): 13.8 (±5.4) 800 IU (*n* = 23): 19.0 (±13.2) *P* > 0.05	Control (*n* = 25): 28.2 (±16.6) 200 IU (*n* = 18): 36.0 (±19.4) 800 IU (*n* = 17): 78.4 (±31.5) ***P*** < **0.001**	Control: 16 (64.0%) 200 IU: 12 (86.0%) 800 IU: 12 (63.0%)	Control: 25 (48.0%) 200 IU: 18 (37.0%) 800 IU: 17 (22.0%) ***P*** = **0.0018**	Control: 12 (38.7%) 200 IU: 4 (21.1%) 800 IU: 6 (26.1%) *P* > 0.05
Ge et al., 2022^c^	No differences between groups at baseline *P* > 0.05	Mean 25(OH)D significantly greater in the 800 IU group vs control group ***P*** < **0.001**	n/a	n/a	Control: 16 (29.0%) 800 IU: 7 (12.3%) ***P*** = **0.028**
Elfarargy et al., 2022^d^	Placebo: 21.0 (±3.7) 800 IU: 20.0 (±3.6) *P* > 0.05	Placebo: 21.5 (±3.8) 800 IU: 39 (±4.3) ***P*** = **0.001**	n/a	n/a	Placebo: 9 (18.0%) 800 IU: 2 (4.0%) ***P*** = **0.025**
Cho et al., 2017^e^	D: 8.3 (±1.9) ND: 21.4 (±8.5) ***P*** < **0.001**	D (*n* = 17): 43.1 (±20.3) ND (*n* = 26): 57.7 (±21.9) ***P*** = **0.03**	Total: 20 (41.0%)	Total: 0 (0%)	Total: 25 (48.0%) D: 7 (37.0%) ND: 16 (55.0%) *P* > 0.05
Bozkurt et al., 2017^f^	400 IU: 16.8 (±6.6) 800 IU: 15.2 (±5.9) 1000 IU: 17.2 (±7.2) *P* = 0.529	400 IU: 29.4 (±13.0) 800 IU: 40.0 (±21.4) 1000 IU: 43.0 (±18.9) 800 IU and 1000 IU vs 400 IU ***P*** = **0.001**	All: 87 (72%) 400 IU: 30 (75.0%) 800 IU: 30 (73.0%) 1000 IU: 27 (68.0%) *P* = 0.738	All: 14 (11.6%) 400 IU: 9 (22.5%) 800 IU: 4 (9.8%) 1000 IU: 1 (2.5%) 1000 IU vs 400 IU RR: 0.09, 95% CI: 0.01–0.74, ***P*** = **0.007**	400 IU: 10 (25.0%) 800 IU: 6 (15.0%) 1000 IU: 11 (27.5%) *P* = 0.367
Natarajan et al., 2014^g^	No differences between groups at baseline *P* = 0.69	Mean 25(OH)D significantly greater in the 800 IU group ***P*** < **0.001**	400 IU: 40 (83%) 800 IU: 37 (79%) *P* = 0.57	400 IU: 30 (67.7%) 800 IU: 16 (38.1%) RR: 0.57 [95% CI: 0.37–0.88], ***P*** = **0.008**	400 IU: 1 (2.0%) 800 IU: 2 (4.0%) *P* = 0.62

Abbreviations: 25(OH)D, 25‐hydroxy vitamin D; BPD, bronchopulmonary dysplasia; D, deficient; IU, international units; ND, not deficient; VDD, vitamin D deficiency.

^a^ Vitamin D deficiency classified as serum 25(OH)D 25(OH)D < 20 ng/ml in all studies except Ge et al., which classified deficiency as serum 25(OH)D 25(OH)D < 10 ng/ml.

^b^ No differences in baseline serum 25(OH)D or VDD; BPD outcomes measured at 28 days as predictive risk of BPD estimated with the Neonatal BPD Estimate Calculator vs actual survival without BPD diagnosis at 36 weeks' PMA or discharge; BPD defined as respiratory support at 36 weeks postmenstrual age classified as grade 1 with nasal cannula ≤ 2 L/min, grade 2 noninvasive ventilation, and grade 3 invasive mechanical ventilation; all cases of BPD listed in table by treatment group, study outcomes reported between group comparisons of BPD diagnosis stratified by severity: no BPD (*P* = 0.36) mild BPD (*P* = 0.73), moderate BPD (*P* = 0.30), and severe BPD (*P* = 0.36).

^c^ Mean serum 25(OH)D depicted in violin graph with *P* value; BPD outcomes at 36 weeks postmenstrual age defined as oxygen therapy of >28 days since birth classified as mild (no oxygen at needed at discharge), moderate (oxygen therapy and FiO2 < 30%), and severe (mechanical ventilation and FiO2 > 30%).

^d^ BPD diagnosed when oxygen needed for ≥28 days and abnormal chest x‑rays with multiple high‐density opacifications.

^e^ All participants received 800 IU vitamin D; groups were stratified at enrollment by vitamin D status at birth as serum 25(OH)D ≥ 10 ng/ml not deficient (ND) or <10 ng/ml deficient (D); BPD diagnosed if supplemental oxygen was needed at 28 days and 36 weeks postmenstrual age; BPD outcomes reported as moderate or severe BPD at 36 weeks postmenstrual age.

^f^ BPD outcomes measured at 36 weeks postmenstrual age; BPD diagnosis not defined; no significant differences in the rate of VDD between the 400 IU and 800 IU group (RR: 0.37, 95% CI: 0.10–1.33, *P* = 0.118).

^g^ BPD outcomes measured at 40 weeks postmenstrual age; BPD diagnosis not defined.

### Literature review

Aristizabal et al.^22^ conducted a secondary analysis of the aforementioned Fort et al.^15^ study to evaluate the safety, efficacy, and predictive risk of BPD associated with different vitamin D doses in premature infants. The parent study randomly assigned 100 preterm infants born ≤28 weeks GA to receive daily enteral placebo (*n* = 36), 200 IU vitamin D (*n* = 34), or 800 IU vitamin D (*n* = 30) for 28 days, starting within 72 h of enteral nutrition (EN).^22^ All participants also received an estimated additional 200 IU of vitamin D/day from parenteral nutrition and EN.^22^ In the secondary analysis, data from 73 infants were analyzed, revealing that the 800 IU group had significantly higher mean serum 25(OH)D levels at 28 days (78.4 ± 31.5 ng/ml), compared with the placebo (28.2 ± 16.6 ng/ml) and 200 IU groups (36.0 ± 19.4 ng/ml, *P* < 0.001).^22^ At birth 54.8% (*n* = 40) of participants had VDD, and by 28 days, the 800 IU group had a significantly lower rate of VDD (22.0%) compared with the placebo (48.0%) and 200 IU groups (37.0%, *P* = 0.0018).^22^ However, there were no significant differences between groups in either the predictive risk of survival without BPD or the actual survival rate without BPD at 36 weeks PMA (*P* = 0.49 and *P* = 0.36, respectively). Although the 200 IU and 800 IU groups had a lower predictive risk of BPD, including severe BPD, compared with placebo, these results were not statistically significant.^22^

Ge et al.^19^ conducted a prospective, unblinded RCT of preterm infants born <32 weeks GA with BW < 1500 g to evaluate the effect of early vitamin D supplementation on BPD prevention. Participants were randomly assigned to the control group (*n* = 55) or the vitamin D–supplement group (*n* = 57) to receive 800 IU of enteral vitamin D per day for 28 days. The investigators did not describe the control intervention, provision of placebo, or estimated vitamin D intake for the control group.^19^ The study found that at 36 weeks corrected age (CA), the 800 IU group had significantly higher serum 25(OH)D levels (*P* < 0.001) and a lower incidence of BPD (12.3% vs 29.0%, *P* = 0.028).^19^

Elfarargy et al.^17^ randomly assigned 100 preterm infants into a prospective, double‐blind RCT to assess the effect of vitamin D supplementation on BPD prevention.^17^ Study participants received a 14‐day intervention of either a placebo (*n* = 50) or 800 IU (*n* = 50) of daily enteral vitamin D supplementation.^17^ By day 14, the 800 IU group had significantly higher serum 25(OH)D levels (39.0 ± 4.3 ng/ml) compared with the control group (21.5 ± 3.8 ng/ml, *P* = 0.001).^17^ Fewer participants in the 800 IU group had confirmed BPD (4.0%, *n* = 2) compared with the placebo group (18.0%, *n* = 9, *P* = 0.025).^17^

Cho et al.^21^ conducted a prospective cohort study enrolling 52 preterm infants with BW < 1500 g to evaluate the safety and efficacy of early administration of daily 800 IU vitamin D supplementation. The intervention started on the 14th day of life with total vitamin D intake from all sources not exceeding 900 IU/day.^21^ Study outcomes consisted of the rate of vitamin D sufficiency (>30 ng/ml), the rate of VDD (<10 ng/ml),^21^ and the rate of moderate or severe BPD at 36 weeks PMA.^21^ At birth, participants with nondeficient cord blood 25(OH)D (ND group) had significantly higher mean serum 25(OH)D (21.4 ± 8.5 ng/ml vs 8.3 ± 1.9 ng/ml, *P* < 0.001) and a greater rate of vitamin D sufficiency (17.0% vs 0.0%, *P* = 0.07) than participants classified as deficient at birth (D group).^21^ The ND group had significantly higher serum 25(OH)D levels (57.7 ± 21.9 ng/ml vs 43.1 ± 20.3 ng/ml, *P* = 0.03) and a higher rate of vitamin D sufficiency (88.0% vs 65.0%, *P* = 0.12) after 800 IU vitamin D supplementation at 36 weeks PMA.^21^ Diagnosis of moderate or severe BPD occurred in 48.0% (*n* = 25) of the total cohort and more participants classified without VDD at birth developed BPD; however, there were no significant differences in the rate of BPD between groups (D group: 55.0% vs ND group: 37.0%, *P* > 0.05).^21^

In a 2017 prospective, unblinded RCT, Bozkurt et al.^20^ randomly assigned 121 preterm infants born ≤32 weeks GA to receive daily vitamin D supplementation of 400 IU (*n* = 40), 800 IU (*n* = 41), or 1000 IU (*n* = 40), starting once EN met 75% of the target volume and continuing through 36 weeks PMA. The intervention did not account for vitamin D intake from fortified human milk or preterm formula (283–320 IU/day and 288–300 IU/day at 160 ml/kg/day, respectively).^20^ Baseline serum 25(OH)D levels and rates of VDD (<20 ng/ml) were similar across groups and by 36 weeks PMA, mean serum 25(OH)D levels were significantly higher in the 800 IU group (40.0 ± 21.4 ng/ml) and the 1000 IU (43.0 ± 18.9 ng/ml) group compared with the 400 IU group (29.4 ± 13.0 ng/ml, *P* = 0.001).^20^ VDD persisted in only 2.5% (*n* = 1) of infants in the 1000 IU group vs 22.5% (*n* = 9) in the 400 IU group (RR: 0.09, 95% CI: 0.01–0.74, *P* = 0.007).^20^ Differences between the 400 IU and 800 IU groups were not statistically significant (*P* = 0.118). Overall BPD incidence was 22.3% (*n* = 27) with no significant differences between groups (400 IU: 25.0%, 800 IU: 15.0%, 1000 IU: 27.5%; *P* = 0.367).^20^

Lastly, Natarajan et al.^18^ conducted a prospective, double‐blinded RCT investigating the effect of 400 IU compared with 800 IU daily vitamin D supplementation on VDD prevalence at 40 weeks PMA among preterm infants born 28–34 weeks GA.^18^ The investigators randomly assigned 87 preterm infants to receive either 400 IU (*n* = 42) or 800 IU (*n* = 45) daily vitamin D for 3 months.^18^ To prevent exceeding the study group allocation, the daily intervention dose was adjusted to account for other sources of vitamin D intake.^18^ Baseline vitamin D status was similar between groups; however, at 40 weeks PMA, the 800 IU group had significantly higher median serum 25(OH)D (22.6 ng/ml vs 15.7 ng/ml, *P* = 0.001) and a lower rate of VDD (38.1% vs 66.7%, RR: 0.57, *P* = 0.008).^18^ BPD was diagnosed in 3.4% (*n* = 3) of the total study population and there was no significant difference in BPD rates between groups (400 IU: 2.0%, 800 IU: 4.0%, *P* = 0.62).^18^

## DISCUSSION

### Vitamin D status

Vitamin D status at birth showed a baseline VDD incidence ranging from 54.8% to 88.5% in three studies in which VDD was defined as serum 25(OH)D < 20 ng/ml^18^, ^20^, ^22^ and 48.8% in Cho et al.^21^ in which VDD was defined as serum 25(OH)D < 10 ng/ml.^21^ Daily 800 IU vitamin D supplementation reduced VDD in two studies.^18^, ^22^ Aristizabal et al.^22^ reported the lowest VDD rate in the 800 IU group compared with the control and 200 IU groups, whereas Natarajan et al.^18^ reported significantly lower rates of VDD in the 800 IU group compared with the 400 IU group. Bozkurt et al.^20^ observed a significant difference in VDD only between the 1000 IU group compared with the 400 IU group, finding no differences between the 800 IU and 400 IU groups.

Across all studies, daily 800 IU vitamin D supplementation significantly increased mean serum 25(OH)D from baseline, and the 800 IU treatment groups had significantly higher mean 25(OH)D levels than their comparison groups.^17^, ^18^, ^19^, ^20^, ^21^, ^22^ Aristizabal et al.^22^ reported that the 800 IU group had significantly higher serum 25(OH)D at day 28 compared with the control and the 200 IU groups. Elfarargy et al.^17^ reported a significant increase in mean serum 25(OH)D after 2 weeks of 800 IU supplementation. Ge et al.,^19^ Bozkurt et al.,^20^ and Natarajan et al.^18^ found that daily 800 IU vitamin D supplementation increased serum 25(OH)D at 36 weeks CA, 36 weeks PMA, or 40 weeks PMA, respectively, compared with the control groups. Bozkurt et al.^20^ also reported greater serum 25(OH)D in the 1000 IU group compared with 400 IU at 36 weeks PMA.^20^

### Vitamin D supplementation and lower BPD incidence

The two studies with BPD as the primary outcome both observed a lower incidence of BPD among the 800 IU intervention groups.^17^, ^19^ BPD incidence among the 800 IU group in Elfarargy et al.^17^ was 4.0% after a 14‐day intervention and 12.3% after a 28‐day intervention in Ge et al.^19^ Although expert guidelines recommend at least 400 IU vitamin D per day for preterm infants,^10^, ^11^ the control groups in both studies received no vitamin D supplementation, which may have contributed to the higher BPD incidence among the control groups.^17^, ^19^ However, in Elfarargy et al.,^17^ the incidence of BPD among the total study population at 11.0% was below the global average.^1^, ^23^ The low incidence of BPD may be attributed to the baseline clinical characteristics of the study population, which had a higher mean GA (33.3 weeks) and mean BW (1914.0 g) compared with the typical preterm population and those at greater risk for BPD,^5^, ^6^, ^23^ thereby limiting the generalizability of the study findings. In contrast, the participants in Ge et al.^19^ had a lower GA (29.2–29.8 weeks) and BW (1240.0–1290.0 g) and the vitamin D intervention was twice as long. Although the overall BPD incidence in Ge et al.^19^ was 20.5%, incidence in the 800 IU group was 12.3% and below the global average,^1^, ^23^ suggesting that higher doses and longer duration of vitamin D supplementation may help prevent BPD.

### Vitamin D supplementation and no difference in BPD incidence

Despite daily 800–1000 IU vitamin D supplementation, four of the six included studies did not demonstrate differences in the incidence of BPD between treatment groups.^18^, ^20^, ^21^, ^22^ Aristizabal et al.^22^ observed that daily vitamin D intake from all sources between 400–1000 IU was associated with a lower predictive risk of BPD; however, the actual incidence of BPD did not differ between treatment groups.^22^ This secondary data analysis of an RCT was not originally designed or powered to evaluate BPD outcomes, and the assigned vitamin D doses were not adjusted for the estimated additional ~200 IU from other sources—factors that may have influenced the study's findings.^22^ Cho et al.^21^ observed a reduction in VDD (25[OH]D > 10 ng/ml) and an increase in vitamin D sufficiency (25[OH]D > 30 ng/ml) with 800 IU supplementation, but the overall BPD was 48%^21^ within the global average incidence range.^1^, ^23^ The study protocol was described as early initiation; however, vitamin D supplementation began on day 14 of life and the effect of earlier intervention in reducing BPD cannot be ruled out.^21^ The lack of a comparison group and insufficient power for BPD outcomes limits conclusions about the impact of vitamin D supplementation on BPD.^21^

In Bozkurt et al.,^20^ no differences in BPD incidence were found between treatment groups and the overall incidence was 22.3%, which was on the lower end of the global average range.^1^, ^23^ This study was not powered for clinical outcomes, such as BPD incidence and the vitamin D dose from EN supplied an additional 283–320 IU/day above the intervention dose.^20^ It can be postulated that the nonsignificant difference in BPD incidence between groups may be attributed to higher cumulative daily vitamin D intake from all sources ranging from 683 to 1320 IU/day, rather than the allocated 400–1000 IU intervention.^20^ Natarajan et al.^18^ reported no difference in BPD incidence between the 400 IU and 800 IU groups, although the 800 IU group had significantly higher mean serum 25(OH)D levels and a lower rate of VDD.^18^ This study was a well‐designed blinded RCT that accounted for all sources of enteral vitamin D and reduced the study dose according to the intervention allocation; however, it too was not powered for clinical outcomes, and the overall BPD incidence was low (3.4%)^18^ and below the global average.^1^, ^23^

### BPD risk factors and adverse outcomes

In evaluating the relationship between vitamin D supplementation and BPD in preterm infants, accounting for postnatal confounding factors must be considered. Known risk factors for BPD, such as younger GA, lower BW, chorioamnionitis, antenatal steroid administration, sepsis, patent ductus arteriosus, duration of mechanical ventilation, and oxygen supplementation, were variably accounted for among the studies reviewed.^24^, ^25^ Ge et al. reported that the 800 IU treatment group had significantly fewer hours of mechanical ventilation (247.4 ± 47.2 vs177.8 ± 42.9, *P* < 0.004) and supplemental oxygen (556.5 ± 132.2 vs 367.5 ± 121.8, *P* < 0.015) compared with the control group receiving no vitamin D supplementation.^19^

However, in contrast, studies such as Elfarargy et al.,^17^ Bozkurt et al.,^20^ and Natarajan et al.^18^ found no differences in days of mechanical ventilation, supplemental oxygen, or nasal continuous positive airway pressure between the control and vitamin D treatment groups. All studies included preterm infants <34 weeks GA and <1500 g except Elfarargy et al.^17^ (mean BW 1913.7 ± 93 g ‐ 1914.2 ± 92 g); however, among all studies, there were no differences between groups baseline GA and BW.^17^, ^18^, ^19^, ^20^, ^21^, ^22^ Between‐group BPD risk factors, such as chorioamnionitis, antenatal steroid administration, sepsis, patent ductus arteriosus, were only reported in four studies, none of which found differences between treatment groups.^18^, ^19^, ^20^, ^21^ Although the studies examined between‐group differences in select BPD risk factors, these variables were not adjusted for in multivariate analysis, leaving the potential for residual confounding.

### Safety of vitamin D supplementation >400 IU/Day

Some clinicians are hesitant to administer higher doses of vitamin D out of concern for toxicity despite expert guidance for intake of up to 1000 IU/day.^13^ The occurrence of vitamin D excess or toxicity was low among the four studies that conducted safety assessments of the treatment dose.^18^, ^20^, ^21^, ^22^ Aristizabal et al.^22^ concluded that a daily 800 IU dose of vitamin D was safe after finding no significant differences in hypercalcemia (serum ionized calcium >5.8 mg/dl) between groups (*P* = 0.13).^22^ Cho et al.^21^ reported three cases of nephrocalcinosis in the 800 IU group; however, only one case also exhibited hypercalciuria (urinary calcium to creatinine ratio [UCa/Cr] < 0.8), which was attributed to vitamin D excess (25(OH)D > 80 ng ml).^21^ Similarly, Bozkurt et al.^20^ reported two cases of vitamin D excess (25(OH)D > 100 ng/ml) of which one case also had nephrocalcinosis; however, the investigators could not confirm if it was due to the vitamin D dose or other causes.^20^ Natarajan et al.^18^ found one case of vitamin D excess (25(OH)D > 100 ng/ml), no occurrences of nephrocalcinosis, and no differences in hypercalciuria (UCa/Cr < 0.8) between treatment groups (*P* = 0.72).^18^

### Strengths and limitations

Since the 2022 Kumar et al.^14^ systemic review and meta‐analysis, only included one study with BPD outcomes, a strength of this literature review was the inclusion of six studies assessing vitamin D supplementation that reported BPD incidence outcomes among preterm infants.^17^, ^18^, ^19^, ^20^, ^21^, ^22^ An additional strength is the RCT design of four of the six included studies,^17^, ^18^, ^19^, ^20^ with two of them specifically aimed to evaluate BPD incidence;^17^, ^19^ however, only Elfarargy et al.^17^ was powered to detect differences in BPD incidence between treatment groups. Limitations include the small sample sizes of each study and that BPD incidence was not the primary outcome of four studies.^17^, ^18^, ^19^, ^20^ Although the majority of the studies included participants at the highest risk of BPD with mean GA < 32 weeks,^19^, ^20^, ^21^, ^22^ the mean GA of the participants in two studies was >32 weeks GA, which may have impacted the BPD incidence in these lower‐risk study populations.^17^, ^18^ Confounding ancillary treatments that impact BPD incidence, such as antenatal steroids and surfactant^6^ were only reported in two studies, both reporting no differences between treatment groups.^19^, ^20^ The vitamin D dose, timing of initiation, and intervention duration varied, which limits comparisons of outcomes between the studies.^17^, ^18^, ^19^, ^20^, ^21^, ^22^ In addition, the generalizability of this literature review is limited by the heterogeneous characteristics between studies in the patient populations, study designs, and vitamin D interventions.

Considering the outcomes of the studies in this review, there appears to be a clinically relevant trend toward lower BPD incidence with daily 800 IU vitamin D supplementation.^17^, ^18^, ^20^, ^21^, ^22^ However, taken collectively, the study results from this literature review limit the ability to identify the exact optimal dose, timing of initiation, and duration of vitamin D supplementation that may reduce or prevent the development of BPD among preterm infants.

## CONCLUSIONS AND IMPLICATIONS

Based on the evidence reviewed, daily 800‐1,000 IU vitamin D supplementation, compared with no supplementation or conventional 400 IU/day, may reduce the incidence of BPD among preterm infants ≤36 weeks of GA. Since the body of evidence on higher doses of vitamin D for outcomes other than vitamin D sufficiency and bone health is limited, more research is needed to determine the optimal daily intake to support immune system modulation, pulmonary maturation, and BPD prevention. Future research should be of RCT design and be powered to detect the risk of developing BPD while controlling for confounders. The study populations should include preterm infants at higher risk for BPD (<32 weeks GA and 1000 g BW)^5^ and the intervention should compare 800–1000 IU vitamin D to the standard 400 IU standard while accounting for all sources of vitamin D.

## AUTHOR CONTRIBUTIONS

Tara Rebele contributed to the conception and design of the research. Corey Hawes, Stephani Johnson, and Melanie Newkirk equally contributed to the design of the research. Tara Rebele contributed to the acquisition and analysis of the data. Tara Rebele contributed to the interpretation of the data. Tara Rebele drafted the article. All authors critically revised the article, agree to be fully accountable for ensuring the integrity and accuracy of the work, and read and approved the final article.

## CONFLICT OF INTEREST STATEMENT

Tara Rebele is employed by BASF Corporation and serves on the Mead Johnson Nutrition Speaker's Bureau. The remaining authors declare no conflicts of interest.
